# Improving health-related quality of life in women with breast, blood, and gynaecological Cancer with an eHealth-enabled 12-week lifestyle intervention: the women’s wellness after Cancer program randomised controlled trial

**DOI:** 10.1186/s12885-022-09797-6

**Published:** 2022-07-08

**Authors:** Charrlotte Seib, Debra Anderson, Amanda McGuire, Janine Porter-Steele, Nicole McDonald, Sarah Balaam, Diksha Sapkota, Alexandra L. McCarthy

**Affiliations:** 1 Menzies Health Institute Queensland and School of Nursing and Midwifery, Griffith University, Queensland, Australia; 2 Faculty of Health, University of Technology Sydney, Sydney, New South Wales Australia; 3 The Wesley Hospital Choices Cancer Support Program, Wesley Hospital, Brisbane, Queensland Australia; 4 Menzies Health Institute Queensland, Griffith University, Queensland, Australia; 5 School of Nursing, Midwifery and Social Work, The University of Queensland, Queensland, Australia; 6 Griffith Criminology Institute, Griffith University, Queensland, Australia; 7 School of Nursing, Midwifery and Social Work, The University of Queensland, and Mater Research Institute, Queensland, Australia

**Keywords:** Cancer, Women, Health-related quality of life, Health behaviour, Intervention

## Abstract

**Background:**

The residual effects of cancer and its treatment can profoundly affect women’s quality of life. This paper presents results from a multisite randomized controlled trial that evaluated the clinical benefits of an e-health enabled health promotion intervention (the Women’s Wellness after Cancer Program or WWACP) on the health-related quality of life of women recovering from cancer treatment.

**Methods:**

Overall, 351 women previously treated for breast, blood or gynaecological cancers were randomly allocated to the intervention (WWACP) or usual care arms. The WWACP comprised a structured 12-week program that included online coaching and an interactive iBook that targeted physical activity, healthy diet, stress and menopause management, sexual wellbeing, smoking cessation, alcohol intake and sleep hygiene. Data were collected via a self-completed electronic survey at baseline (t_0_), 12 weeks (post-intervention, t_1_) and 24 weeks (to assess sustained behaviour change, t_2_). The primary outcome, health-related quality of life (HRQoL), was measured using the Short Form Health Survey (SF-36).

**Results:**

Following the 12-week lifestyle program, intervention group participants reported statistically significant improvements in general health, bodily pain, vitality, and global physical and mental health scores. Improvements were also noted in the control group across several HRQoL domains, though the magnitude of change was less.

**Conclusions:**

The WWACP was associated with improved HRQoL in women previously treated for blood, breast, and gynaecological cancers. Given how the synergy of different lifestyle factors influence health behaviour, interventions accounting for the reciprocity of multiple health behaviours like the WWACP, have real potential for immediate and sustainable change.

**Trial registration:**

The protocol for this randomised controlled trial was submitted to the Australian and New Zealand Clinical Trials Registry on 15/07/2014 and approved on 28/07/2014 (ACTRN12614000800628).

**Supplementary Information:**

The online version contains supplementary material available at 10.1186/s12885-022-09797-6.

## Background

Aging populations and the increased prevalence of other cancer risk factors have led to an increased incidence of cancer in women globally [1]. According to the International Agency for Research on Cancer (IARC), more than 4.2 million women worldwide were diagnosed with breast, blood, or gynaecological cancers in 2020, accounting for one-third of new cancers during that period [1, 2]. Cancer incidence in Australia reflects global trends. In 2019, incident cases of breast, blood and gynaecological cancer were estimated to be approximately 32,000 [3]. While cancer rates continue to grow, 5-year survival has also increased. In 2016 it was estimated that two-thirds of Australian women previously diagnosed with cancer were currently living with its long-term effects [3].

Cancer treatments often leave women with a range of residual physical and psychological side effects including neuropathy [4], fatigue, cognitive disruption [5], lymphoedema, osteoporosis [6, 7], treatment-induced menopausal symptoms and psychological distress [8]. These residual effects can compromise women’s ability to sustain healthy lifestyle behaviours [9] and further heighten their risk of treatment-related chronic disease [10]. These residual treatment effects can also profoundly undermine women’s quality of life and physical functioning as they move into older adulthood.

Comprehensive cancer rehabilitation can reduce symptom burden and health service utilisation, whilst generally improving health-related quality of life (HRQOL) [11, 12]. However, for maximum benefits it is critical that supportive interventions address concurrent and overlapping risk factors [7] (not just one risk factor) and also enhance women’s capacity to self-manage any issues in the longer term. This synergistic approach enhances intervention effectiveness and longer-term sustainability [13–15].

While there is international recognition that comprehensive recovery care is needed, services in Australia are rarely resourced to deliver this [7, 16–18]. Once women have completed active treatment, opportunities to access education and support to help them optimise health behaviours are limited [17]. This is further compounded by remoteness, with almost one-third of Australian women living outside major metropolitan areas, and for these women, their restricted access to health services is associated with poorer health outcomes [19]. Thus, while women are living longer after cancer, the opportunity for better recovery is limited, depriving women of the ability to maximise their health potential.

The Women’s Wellness after Cancer Program (WWACP) is a multimodal, individualised, and digitised lifestyle intervention that was designed to address these gaps [20]. The WWACP capitalised on women’s propensity for lifestyle change at the completion of active treatment for breast, gynaecological, or blood cancers. The program, created for the post-acute cancer care milieu, aimed to enhance HRQOL, decrease the late effects of cancer treatment, and reduce chronic disease risk factors in this population. We used an e-health platform to maximise opportunities for engagement while reducing the potential barriers associated with geography, transportation, cost, and time. The primary objective of the study was to explore the effect of the WWACP on the HRQOL of women diagnosed with cancers associated with treatment-induced menopause. It was hypothesised that, compared to controls, women enrolled in the WWACP would report better HRQOL at the end of intervention (Week 12), which would persist at Week 24.

## Methods

### Study design

This multi-centre, single-blinded, randomised controlled 12-week trial included five hospitals in three Australian states, consumer groups, and supportive cancer care services serving women across Australia. The primary aim of this study was to test the efficacy of a multimodal, digitised lifestyle intervention on HRQOL of women treated for cancer. After baseline assessment, three hundred and fifty-one women previously treated for breast, blood or gynaecological cancer were randomly assigned to either an intervention or usual care arm using permuted-block randomisation. A computer-generated allocation sequence [21, 22] using blocks of varying length was developed by the trial statistician, and randomization was performed by the trial coordinator, who logged into a secure server to obtain the next allocation. While blinding of participants was not possible, the trial statistician and study staff (except for the trial coordinator and those who delivered the intervention) were unaware of group allocation.

Data were collected from participants via online questionnaires and virtual consultations at three time points, baseline (*t*_*0*_), 12 weeks (*t*_*1*_) and 24 weeks (*t*_*2*_). The protocol for this trial was submitted to the Australian and New Zealand Clinical Trials Registry on 15/07/2014 (ACTRN12614000800628). Complete protocol details including the funding source, ethical approvals, the sampling and recruitment, randomisation procedure, intervention content and delivery mode, primary and secondary endpoints, and approach to data analysis are described elsewhere [20]. However, some deviations from the reported protocol in relation to time since diagnosis should be noted, with around 22% (*n* = 62) of women enrolled in the study being diagnosed with cancer more than 2 years earlier.

### Study population

Women who had completed treatment for breast, blood, or gynaecological cancer who were proficient in English, and who had access to an Apple computer and/or iPad were invited to participate in the study. Most participants reported having combined cancer treatment (55.9% reported surgery, chemotherapy + radiation; 20.6% reported surgery + radiation; 12.5% reported surgery + chemotherapy), 76.5% of women’s treatments included hormone therapy, and a smaller proportion reported a single modality treatment (> 1% had radiation therapy and 10% of participants reported having surgery). Women with metastatic or advanced cancer, inoperable or active loco-regional disease, or on maintenance chemotherapy for blood cancers were not eligible to participate in this study (they are the focus of future intervention studies).

### Intervention

The WWACP was underpinned by Social Cognitive Theory, an approach that recognises the importance of reciprocal determinism and behavioural capacity on self-efficacy for health behaviour change [23]. The program supported women to make incremental and feasible changes to less healthy lifestyle behaviours, enhancing their self-efficacy and developing and sustaining healthy lifestyle habits. The intervention was delivered via an e-enabled platform including an *iBook* and virtual health consultations (detailed previously [20]). Briefly, the structured 12-week intervention comprised a *website* with educational podcasts and exercise planners; an interactive *iBook* with practical information to support adoption and maintenance of healthy lifestyle behaviours and tracking of health behaviour changes goals; three *virtual consultations* with a registered nurse to support the development of realistic and achievable health goals and explore the strategies to enhance women’s self-efficacy for health behaviour change.

### Primary endpoint

Initially, we measured HRQoL using two instruments, the Functional Assessment of Cancer Therapy—General (FACT—G) scale [24] and the Short Form Health Survey (SF-36) [25]. However, while the FACT—G is a robust and well-validated measure for evaluating HRQoL among patients receiving cancer treatment [24, 26, 27], its responsiveness in ‘longer-term’ after cancer groups in largely unknown [26]. As almost one-quarter of women in this study were more than 2 years since diagnosis, and the focus of the FACT—G instrument is on measuring cancer-specific concerns rather than broader HRQoL concerns reported in after-cancer populations [26], this paper reports SF-36 data only.

The SF-36 is a 36-item self-reported generic health measurement that examines eight dimensions of health, including physical functioning (PF), role limitations due to physical health (RP), bodily pain (BP), general health (GH), vitality (VT), social functioning (SF), role limitations due to emotional health (RE), and mental health (MH). The instrument also provides two composite measures for mental (Mental Component Summary, MCS) and physical (Physical Health Component, PCS) functioning and wellbeing [25]. The SF-36 is scored using QualityMetric Health Outcomes™ Scoring Software (QualityMetric, Inc., Lincoln, RI) to form standardised 100-point scales, with higher scores denoting better physical and mental functioning [25].This instrument has been extensively used in a variety of clinical and community populations, including women after cancer treatment, with consistently good reliability and validity [25, 28].

### Statistical analysis

Statistical analyses were performed using Statistical Package for the Social Sciences, version 23 (SPSS, Inc., Chicago, IL) and STATA 11 (StataCorp, Inc., College Station, TX). SPSS was used for generating descriptive (are expressed as counts and percentages, mean, and standard deviation (SD) and bivariate statistics (t-tests), and one-way Analysis of Covariance (ANCOVA). Statistical significance set at α = 0.05.

To assess the potential impact of attrition on results, two separate analyses, per-protocol (PP) and intent-to-treat (ITT), were performed in SPSS [29]. ITT analysis imputed outcome data for 12-weeks (*t*_*1*_) and 24-weeks (*t*_*2*_) using the last-observation-carried-forward method of imputation.

**Fig. 1:**
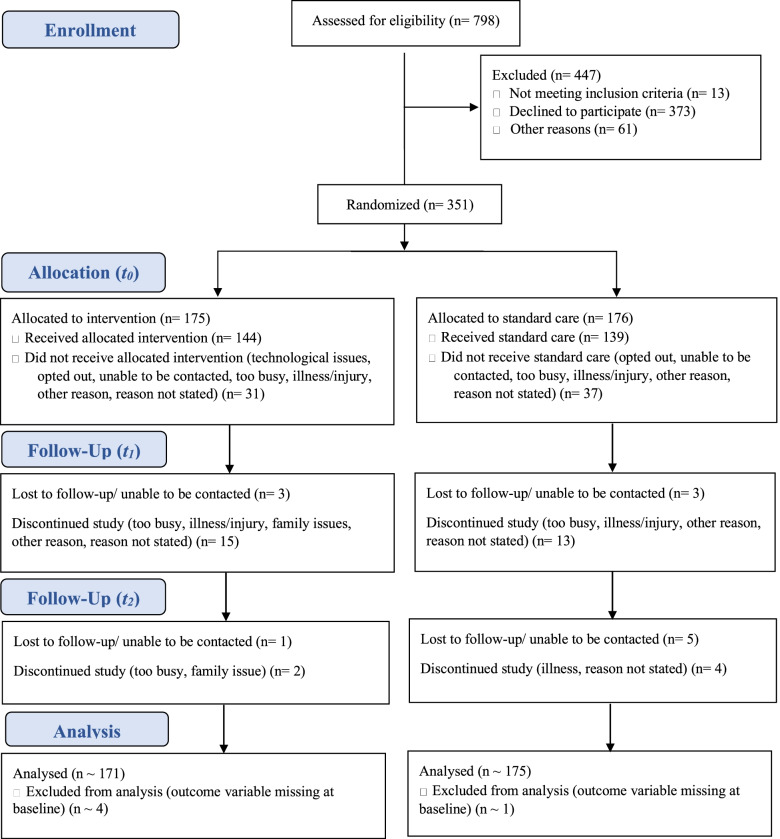
Consort diagram of the Women’s Wellness after Cancer Program (WWACP) clinical trial. All participants provided baseline data (*t0*) before being randomised to either the intervention or standard care group. The intervention group completed a 12-week e-enabled lifestyle intervention while the standard care group received general information only. Data were collected from all participants at 12- (*t1*) and 24- weeks (*t2*)

Incomplete baseline scores were noted in several instances with five of the women enrolled in the study did not provide sufficient baseline data to estimate the primary endpoints and were excluded from the analysis (discussed further in Fig. 1 and Table S1). Withdrawal from the study was greatest among participants who completed the online questionnaire but who did not also want to provide further biophysical data via a virtual consultation. The characteristics of the 68 women lost to follow-up (LTFU) during this period were compared with women who continued in the study (see Table S2). Findings suggested that women who withdrew were more likely to be in the lower income brackets (*p* = 0.01), born in Australia (*p* < 0.01), but were more likely to speak a language other than English at home (*p* = 0.03).

Within-group change in HRQoL scores over the intervention period were assessed using split sample paired t-tests and Cohen’s *d.* Effect size was derived using an online calculator that accounted for correlations in the outcome variable over time (more information can be obtained at https://memory.psych.mun.ca/models/stats/effect_size.shtml), which suggested that *d* = 0.2, 0.5 or 0.8 were equivalent to a small, moderate, and large effect respectively [30].

Individual- and group-level changes in HRQoL across the trial period using linear mixed-effect models (LMM) with an autoregressive residual variance–covariance structure (AR 1) [31] were examined in STATA. This structure was chosen because of the homogeneous variances, decreasing correlations with distance (*t*_*0*_*, t*_*1*_*, t*_*2*_), and the most favourable fit statistic (i.e., the smallest Akaike Information Criterion [AIC] value). To determine model fit, AIC was used in addition to a likelihood ratio *chi*-square test (LR test). The LR test statistic generates a *chi*-squared value which, if statistically significant, suggests that the less restrictive model (i.e., the random intercept and slope model) is significantly better than the random intercept only (more restrictive) model.

## Results

A total of 798 patients were assessed for eligibility for the study, of whom 351 consented to participate and were randomised following baseline data collection. Among those who commenced the trial, 18 women from the intervention group and 16 women from the control group did not provide data at 12 weeks. A further 12 women were unavailable at 24 weeks (3 intervention participants and 9 control participants). Figure 1 provides an overview of recruitment and flow through the study.

Table 1 details the sociodemographic characteristics or cancer variables of study participants at baseline. The average age of participants was 53 years (SD = 8.8) and almost all had been diagnosed with breast cancer (94.7%) around 19 months (SD = 13.4) before commencing the study. Three quarters were married (76.9%) with a small number reporting being separated or divorced (11.3%), widowed (2.6%) or single (9.2%). Over half reported having a university degree (57.5%), around one-quarter (22.8%) had a technical certificate or diploma and a small proportion of the sample had either a junior (≤ Year 10, 9.0%) or senior (Year 12, 10.7%) certificate. Most of the sample were employed in either a full- or part- time capacity (47.8% and 38.2% respectively) before cancer diagnosis and two-thirds (64.6%) reported a gross household income over $80,000 AUD.

**Table 1:** Baseline characteristics of women of the study sample^a^

	Intervention	Control	Total
	n (%) or M (SD)	n (%) or M (SD)	n (%) or M (SD)
Background characteristics			
Mean age (SD)	52.6 (9.4)	53.7 (8.1)	53.2 (8.8)
Marital status			
Married/de facto relationship	133 (77.3)	133 (76.4)	266 (76.9)
Separated or divorced	16 (9.3)	23 (13.2)	39 (11.3)
Widowed	3 (1.7)	6 (3.4)	9 (2.6)
Single	20 (11.6)	12 (6.9)	32 (9.2)
Country of birth			
Australia	121 (69.9)	121 (69.5)	242 (69.7)
Elsewhere	52 (30.1)	53 (30.5)	105 (30.3)
Highest educational attainment			
Year 10 or less (junior school)	13 (7.6)	18 (10.3)	31 (9.0)
Year 11 or 12 (senior school)	17 (9.9)	20 (11.5)	37 (10.7)
Technical certificate/diploma	42 (24.4)	37 (21.3)	79 (22.8)
University degree/postgraduate	100 (58.1)	99 (56.9)	199 (57.5)
Employment status			
Employed (full-time)	75 (47.2)	79 (48.5)	154 (47.8)
Employed (part-time)	62 (39.0)	61 (37.4)	123 (38.2)
Retired	17 (10.7)	16 (9.8)	33 (10.2)
Other	5 (3.1)	7 (3.3)	12 (3.7)
Gross household income			
Less than $20,000 AUD	3 (1.8)	3 (1.7)	6 (1.7)
$20,000—$80,000 AUD	52 (30.4)	48 (27.6)	100 (29.0)
Above $80,000 AUD	109 (63.7)	114 (65.5)	223 (64.6)
Don’t Know	7 (4.1)	9 (5.2)	16 (4.6)
Cancer experience			
Cancer type			
Breast	136 (95.1)	130 (94.2)	266 (94.7)
Gynaecology	2 (1.4)	6 (4.3)	8 (2.8)
Blood	5 (3.5)	2 (1.4)	7 (2.5)
Mean months since diagnosis (SD)	19.3 (14.7)	18.8 (11.9)	19.1 (13.4)

*n* number of participants per group**,***M* mean value**,***SD* standard deviation of the mean value**,***AUD* Australian dollars

^a^Overall n’s might differ because of missing data

Both between-group differences, and within-group changes, in SF-36 domain and composite scores were examined using per-protocol (PP) and intent-to-treat (ITT) analyses. Results suggested that while the magnitude of difference was reduced in some instances in the ITT analysis, all differences remained significant and therefore per-protocol results are presented here (ITT analyses are presented in Tables S3 and S4). Between-group changes in HRQoL scores are presented in Table 2. Following adjustment for baseline scores, a 3-point improvement in role limitations due to physical health (95% CI 0.11 – 5.66, *p* = 0.03) and 2-point improvement in vitality (95% CI 0.36 – 4.13, *p* = 0.02) was noted at 12-weeks and these changes were sustained at 24-weeks *F(1, 238)* = 4.41, *p* = 0.04 and *F(1, 231)* = 4.20, *p* = 0.04 respectively). Similar improvements were noted for bodily pain and PCS scores at 12-weeks (*F(1, 241)* = 5.11, *p* = 0.02; *F(1, 239)* = 7.34, *p* =  < 0.01) though the magnitude of this improvement was reduced at 24-weeks. In contrast, both mental health and general health domain scores showed little change at 12-weeks but a 2-point improvement at 24-weeks (95% CI 0.41 – 4.16, *p* = 0.02 and 95% CI 0.54 – 4.20, *p* = 0.01 respectively). Finally, control group participants reported an average 3.78-point improvement improvements in role limitations due to emotional health at 12-weeks (95% CI 0.98 – 5.78, *p* < 0.01) though this difference was no longer significant at 24-weeks. The results are provided in detail in Table 2.

**Table 2:** Between-group differences in SF-36 domain and composite summary scores of study participants^a^

	Baseline (*t*_*0*_)		Post-intervention (*t*_*1*_)		Follow-up (*t*_*2*_)	
	Intervention (*n* = 175)	Control (*n* = 176)	Intervention (*n* = 120)	Control (*n* = 126)	Intervention (*n* = 123)	Control (*n* = 120)
	M (SD)	M (SD)	M (SD)	M (SD)	M (SD)	M (SD)
Physical functioning (PF)	47.0 (8.2)	45.0 (8.9)	49.6 (7.8)	47.5 (8.1)	50.6 (7.6)	47.4 (9.3)
Role limitations/physical health (RP)	44.7 (11.6)	42.1 (12.0)	50.0 (9.7)	45.3 (11.9)*	48.6 (10.3)	44.7 (12.4)*
Bodily pain (BP)	48.9 (9.0)	47.0 (8.9)	50.8 (9.0)	47.7 (9.5)*	50.2 (9.3)	47.5 (10.2)
General health (GH)	48.0 (8.3)	47.3 (9.5)	48.9 (8.4)	46.8 (8.9)	50.1 (8.8)	47.0 (9.8)*
Vitality (VT)	47.2 (8.8)	46.3 (9.8)	50.5 (8.7)	47.8 (9.4)*	50.5 (9.2)	47.9 (10.0)*
Social Functioning (SF)	46.5 (9.2)	44.9 (10.4)	49.9 (7.9)	47.9 (10.0)	50.1 (8.3)	48.0 (10.6)
Role limitations/emotional health (RE)	45.5 (12.1)	45.5 (12.2)	47.2 (10.9)	49.7 (10.1)**	48.4 (10.5)	47.1 (12.2)
Mental health (MH)	45.1 (9.1)	45.4 (9.4)	42.5 (8.6)	41.8 (9.1)	50.2 (8.5)	48.8 (10.4)*
Physical component summary (PCS)	47.9 (8.7)	45.3 (9.5)	52.0 (8.6)	47.6 (9.2)**	50.2 (8.9)	46.3 (10.4)
Mental component summary (MCS)	45.5 (10.3)	46.1 (10.8)	45.3 (9.7)	46.2 (9.9)	49.5 (9.4)	48.6 (11.6)

Between-group differences at *t*_*1*_ and *t*_*2*_ used one-way ANCOVA adjusting for baseline scores * *p* < .05, ** *p* < .01

*M* mean value, *SD* standard deviation of the mean value, *SF-36* Short Form 36

^a^Overall n’s might differ because of missing data

Within-group changes were also examined over time (Table 3). Overall, women in the intervention group reported modest improvements in many HRQoL measures at 12 weeks and in many instances these improvements were sustained at 24 weeks. For example, a moderate effect was seen in role limitations due to physical health (RP, *d* = 0.45), social functioning (SF, *d* = 0.40), and vitality (VT, d = 0.37) and these improvements were sustained at follow-up. Moreover, while MH scores decreased at 12 weeks, significant improvements were noted at 24 weeks (M_change_*t*_*1*_ = 2.7, *t*(116) = 3.63, *p* < 0.01; M_change_*t*_*2*_ = -8.3, *t*(113) = -13.65, *p* < 0.01). This was also evident in the large effect in mental component summary (MCS) scores at 24 weeks (*d* = 0.63). Similar trends were also detected in the RP, SF, and MH scores of participants in the control group except for role limitations due to emotional health (RE). While women in the intervention group noted little change in RE scores, women in the control group reported a moderate effect at 12 weeks (*d* = 0.42).

**Table 3:** Within-group changes in SF-36 domain and composite summary scores over time using per protocol analysis^a^

	n	M (SD)			Cohen’s d^b^	
	*p*_*1*_*/p*_*2*_	*t*_*0*_	*t*_*1*_	*t*_*2*_	*d*_*1*_	*d*_*2*_
***Intervention group***						
Physical functioning (PF)	119/115	47.9 (7.3)	49.5 (7.8)*	50.8 (7.6)*	0.20	0.24
Role limitations/physical health (RP)	115/113	45.0 (11.3)	49.8 (9.8)**	48.9 (10.2)	0.45	-0.10
Bodily pain (BP)	119/115	48.9 (8.9)	50.9 (9.1)*	50.3 (9.3)	0.22	-0.08
General health (GH)	119/115	48.7 (8.1)	48.8 (8.8)	50.5 (8.8)	0.02	0.23
Vitality (VT)	118/114	47.2 (8.7)	50.5 (8.8)**	51.0 (9.0)	0.37	0.08
Social Functioning (SF)	119/115	46.6 (8.9)	49.8 (7.9)**	50.4 (8.1)	0.40	0.08
Role limitations/emotional health (RE)	117/114	46.9 (11.3)	47.1 (11.0)	48.9 (10.2)	0.01	0.15
Mental health (MH)	117/114	45.3 (8.5)	42.6 (8.6)**	50.8 (8.4)**	-0.34	1.26
Physical component summary (PCS)	117/114	48.2 (8.1)	51.9 (8.6)**	50.2 (9.0)**	0.49	-0.27
Mental component summary (MCS)	116/114	46.0 (9.8)	45.2 (9.8)	50.2 (9.1)**	-0.08	0.63
***Control group***						
Physical functioning (PF)	125/116	45.3 (8.9)	47.5 (8.1)**	47.7 (9.1)	0.32	0.04
Role limitations/physical health (RP)	123/114	41.2 (11.7)	45.4 (11.8)**	44.8 (12.4)	0.34	-0.05
Bodily pain (BP)	125/116	47.3 (8.9)	47.7 (9.6)	47.4 (10.0)	0.05	-0.03
General health (GH)	125/116	47.8 (9.3)	46.8 (8.9)	47.0 (9.7)	-0.15	0.04
Vitality (VT)	125/116	46.6 (9.4)	47.9 (9.4)	48.0 (10.0)	0.16	0.02
Social Functioning (SF)	125/116	44.9 (10.4)	47.9 (10.0)**	48.1 (10.4)	0.33	0.02
Role limitations/emotional health (RE)	121/114	44.9 (12.5)	49.6 (10.1)**	47.4 (12.1)	0.42	-0.19
Mental health (MH)	125/116	46.1 (8.9)	41.9 (9.0)**	49.0 (10.1)**	-0.58	0.91
Physical component summary (PCS)	125/116	45.3 (9.7)	47.6 (9.3)**	46.4 (10.4)	0.29	-0.18
Mental component summary (MCS)	125/116	46.2 (10.9)	46.3 (9.8)	48.9 (11.4)**	0.01	0.27

p_1_, Pair 1 (t_0_ vs. t_1_); p_2_, Pair 2 (t_1_ vs. t_2_); *M* mean value, *SD* standard deviation of the mean value; d_1_, effect size for pair 1; d_2_, effect size for pair 2; *SF-36* Short Form 36

^a^Split sample paired sample t–tests

^b^Cohen’s d effect size accounting for the correlation between outcome variables over time

^*^p < .05

^**^p < .01

Linear mixed effect models (LMM) examined within-group changes, and between-group differences, in health-related quality of life variables over the intervention period. Table 4 shows the results of the best fitting models for the SF-36 domain sub-scales and composite summary scores, while comparative fit statistics are presented in the online supplementary materials (see Table S5). Overall, the less restrictive model (i.e., the random intercept and slope model) provided the best fit for the data, with 25–64% of the variance in HRQoL scores attributable to differences between individuals (RP, *τ* = 0.251; PCS, *τ* = 0.370; PF, *τ* = 0.395; SF, *τ* = 0.512; MH, *τ* = 0.635; MCS, *τ* = 0.524).

**Table 4:** Best fitting linear mixed effect model for adjusted SF-36 domain and composite summary scores^a^

		Group	Simple effects for Control group		Simple effects for the Intervention Group		Random effect^b^	
	Constant	Intervention	*t*_*1*_	*t*_*2*_	*t*_*1*_	*t*_*2*_		
	*β (se)*	*β (se)*	*β (se)*	*β (se)*	*β (se)*	*β (se)*	*β (se)*	*τ*
PF	47.13 (3.39)^**^	1.92 (0.99)	2.41 (0.59)^**^	2.73 (0.73)^**^	1.92 (0.60)^**^	3.03 (0.71)^**^	5.16 (0.82)	.395
RP	42.03 (4.34)^**^	3.92 (1.36) ^**^	4.29 (0.99)^**^	3.47 (1.17)^**^	5.01 (1.01)^**^	3.72 (1.15)^**^	5.61 (1.26)	.251
BP	48.03 (3.75)^**^	1.96 (1.12)	0.50 (0.72)	0.55 (0.82)	2.09 (0.73)^**^	1.33 (0.81)	6.44 (0.57)	.490
GH	44.31 (3.73)^**^	0.69 (1.07)	-0.79 (0.61)	-0.55 (0.68)	0.30 (0.62)	1.59 (0.67)^*^	6.92 (0.44)	.618
VT	43.27 (3.77)^**^	0.68 (1.11)	1.42 (0.72)	1.68 (0.77)^*^	3.16 (0.73)^**^	3.28 (0.76)^**^	6.89 (0.45)	.565
SF	44.62 (3.86)^**^	1.93 (1.15)	3.23 (0.76)^**^	3.61 (0.84)^**^	3.25 (0.77)^**^	3.78 (0.82)^**^	6.78 (0.52)	.512
RE	45.29 (4.30 ^**^	0.91 (1.37)	4.82 (1.07)^**^	2.45 (1.16)^*^	0.60 (1.07)	2.26 (1.13)^*^	6.64 (0.73)	.348
MH	43.50 (3.88)^**^	-0.45 (1.12)	-4.06 (0.68)^**^	3.15 (0.72)^**^	-2.73 (0.69)^**^	5.24 (0.71)^**^	7.37 (0.43)	.635
PCS	46.15 (3.82)^**^	2.89 (1.12)^*^	2.46 (0.65)^**^	1.27 (0.81)	3.91 (0.66)^**^	1.95 (0.79)^*^	5.61 (1.09)	.370
MCS	43.66 (4.24)^**^	-0.25 (1.26)	0.19 (0.80)	2.85 (0.90)^**^	-0.78 (0.82)	3.91 (0.89)^**^	7.48 (0.58)	.524

*t*_*1*_, 12-weeks (post-intervention); *t*_*2*_, 24-weeks (follow-up); β, regression coefficient value; se, standard error; τ, variance partition coefficient accounting for individual variation; *PF* Physical Functioning, *RP* Role limitations due to physical health, *BP* Bodily Pain, *GH* General Health, *VT* Vitality, *SF* Social Functioning, *RE* Role limitations due to emotional health, *MH* Mental Health, *PCS* Physical Component Summary, *MCS* Mental Component Summary

^a^All models adjusted for age, education, marital status, and cancer type

^b^Random effect component accounted for individual variation

^*^p < .05

^**^p < .01

Over the intervention period, some general improvements in HRQoL were noted for both the intervention and control group. More specifically, both groups reported significant improvements in role functioning (PF), role limitations due to physical health (RP), and social functioning (SF) domains (*p* < 0.01 for all). Similar trends were also seen in physical and mental component summary (PCS and MCS) scores, although the magnitude of improvement was smaller.

In contrast, the intervention group reported a significant reduction in limitations associated with bodily pain at Time 1 *(β* = 2.09, *SE* = 0.73,* p* < 0.01) and improvements in general health at Time 2 (*β* = 2.09, *SE* = 0.95, *p* = 0.02) compared to the control group. For both bodily pain and general health domains, around half of the variance in scores was associated with individual difference (*ρ* = 0.490 and *ρ* = 0.618 respectively).

**Fig. 2:**
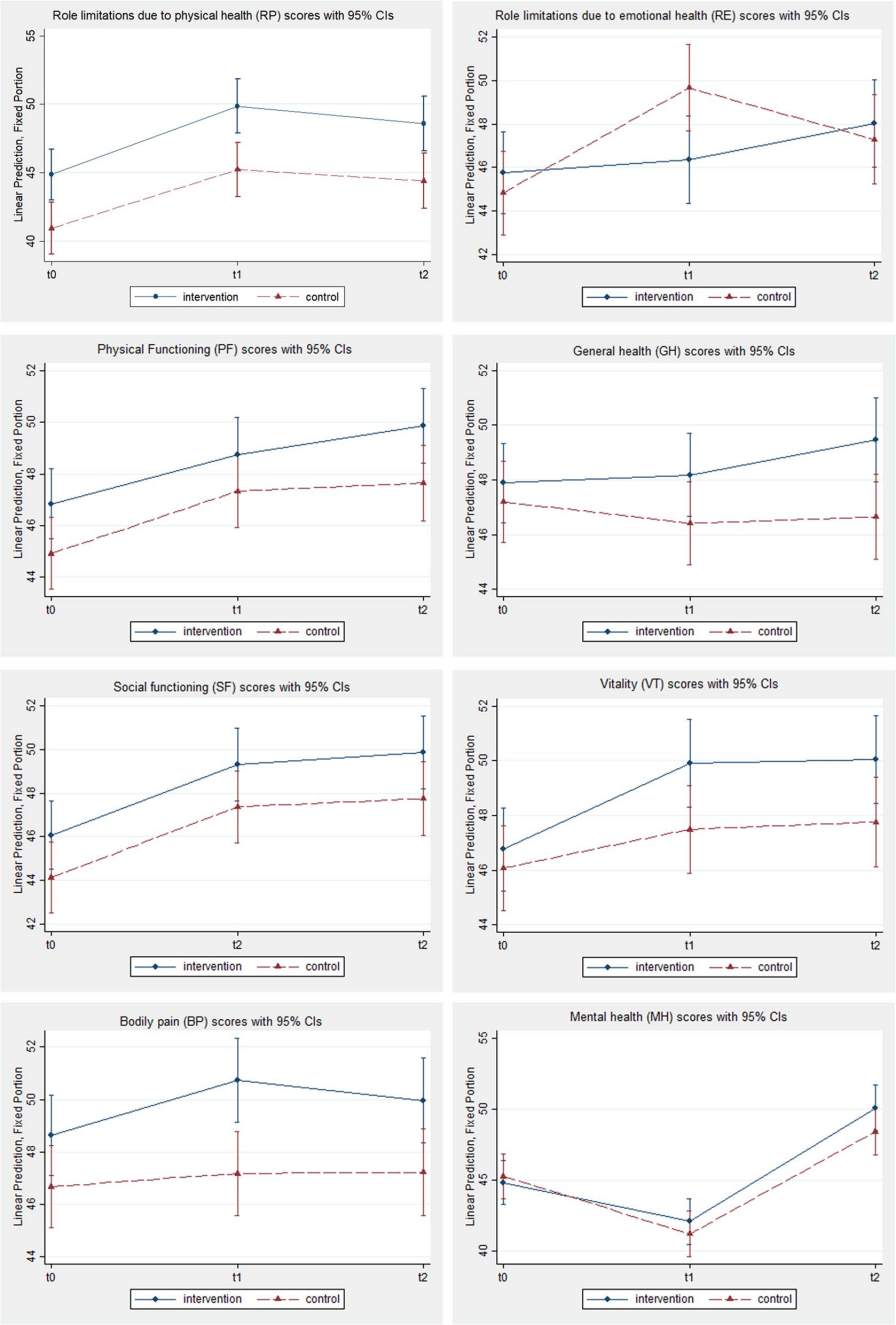
Change over time in SF-36 domain scores

**Fig. 3:**
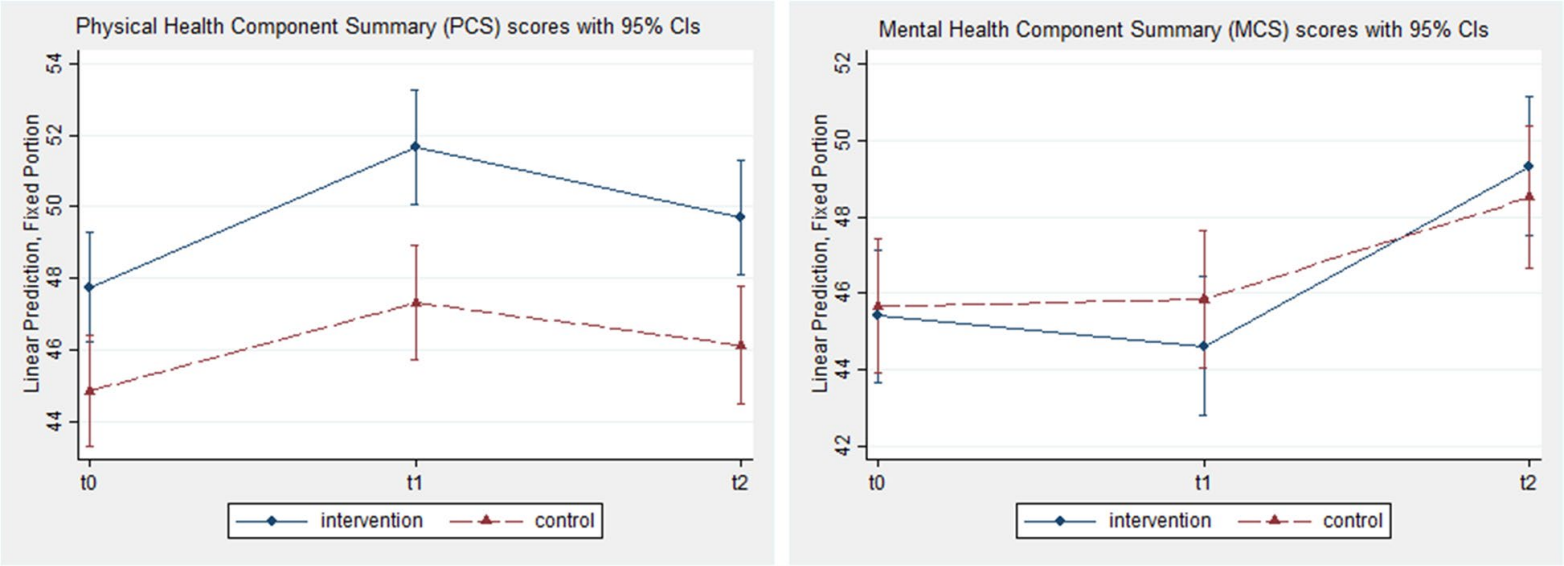
Change over time in PCS and MCS scores

Vitality improved in both groups. In the intervention group an average 3.16- and 3.28-point increase in vitality was detected at Times 1 and 2 respectively. The control group also reported general increases over the same period, albeit to a lesser extent (*t*_*2*_, *β* = 1.68, *SE* = 0.77, *p* = 0.03). Changes in the mental health domain scores were also reported. Notably, at Time 1, significant decrements in mental health scores were detected among participants in both the intervention (*β* = -2.73, *SE* = 0.69, *p* < 0.01) and control (*β* = -4.06, *SE* = 0.68, *p* < 0.01) group. At Time 2 however, these decrements were no longer present, with both groups reporting increases in summary mental health scores. While both groups reported improvements, the magnitude of change differed. The intervention group reported better overall mental health scores at Time 2 compared with control group participants (*β* = 5.24, *SE* = 0.71, *p* < 0.01; *β* = 3.14, *SE* = 0.72, *p* < 0.01 respectively). Linear predictors for all domain scores and component summary scores are presented in Figs. 2 and 3.

## Discussion

The completion of acute cancer treatment is a time of increased vulnerability, as women look for ways to mitigate the risk of cancer recurrence [32, 33] and recover from the effects of treatment. Yet cancer services are necessarily acuity focused. Human and financial resource constraints restrict their capacity to provide the comprehensive recovery care that could optimise women’s health and wellbeing once they complete intensive treatment [7, 16–18]. This multimodal lifestyle intervention was designed to capitalise on this period of heightened receptivity to lifestyle changes, thereby maximising the uptake of healthy behaviours in women after cancer treatment in the absence of other services.

Our results indicate improvements in many HRQoL domains and in component summary scores. Particularly notable were the improvements in general health and bodily pain, vitality, mental health, and global physical and mental health summary scores among women in the intervention group. While improvements in some of these domains (i.e., VT, MH, MCS, PCS) were also detected in the control group, the magnitude of effect in the intervention group was almost double that reported by control group participants. Previous lifestyle interventions in women after cancer have also shown less fatigue, improved HRQoL, and better quality of sleep in the intervention group compared with controls [34, 35]. Taken together, this suggests that lifestyle interventions can provide non-pharmacologic recovery support that optimises quality of life and reduces symptom burden in women previously treated for cancer.

Quality of life, the primary outcome of this study, was identified in our scoping work with the target group as the most important outcome to them [36]. In the exploratory work undertaken to develop the WWACP, participants emphasised that quality of life is not a single factor, it is multidimensional [36]. They also clearly described how the health behaviours that influenced quality of life worked in synergy and operated across physical, emotional, and social domains [36]. In women treated for cancer, healthy behaviours such as good nutrition and physical activity interact constantly in daily life with each other and with psychosocial factors like emotional distress, sleep quality and fear of recurrence. Hence, the WWACP was designed as a bundled intervention to account for the complexity of health behaviour within the context of each participant’s sociocultural milieu and individual goals for wellbeing. This lifestyle intervention accounted for the reciprocity of these factors and as demonstrated in our results, yielded an immediate and sustainable quality of life benefit. Interventions that compartmentalise rather than synergise health behaviours, and which are not underpinned by strong elements of psychosocial support and self-management, often have good results at the end of the intervention period—but these results are rarely sustained at follow-up [13–15, 37]). This observation is reinforced by others who have demonstrated continued adherence and positive patient experiences from multidimensional interventions for weight, diet, and exercise in women with breast cancer [37].

This study has several limitations that should be noted when interpreting the findings. Like many RCTs, selection bias (motivated individuals enrolling in lifestyle intervention) and attrition might have affected the representativeness of the sample. In this study, the largest proportion of women lost to follow-up (LTFU) occurred between randomisation and commencing the study. Excess participant burden associated with the length of the questionnaire and the dual modes of data collection might explain the 20% attrition at this time, though we do not believe it impacted the study outcomes. This study also relied on self-report which is associated with an increased risk of response bias due to the misinterpretation of questions and social-desirability bias even if the survey was de-identified [38]. To minimise the response bias, standard and validated data collection tools were used, and trained data collectors were employed. Finally, most participants were treated for breast cancer, so we hesitate to generalise these findings to women with other cancers. Future research should explore the way in which the core elements of the program can be applied to other cancer groups.

Despite these limitations, this study addresses an unmet need for many women whose cancer-related health effects, while potentially deleterious to their quality of life, are frequently amenable to lifestyle modification. As such, programs like the WWACP have the potential to optimize health by focussing on effective symptom management, preventing late effects, reducing co-morbid conditions, and general health promotion [39]. Moreover, the use of an interactive eHealth platform to deliver a real-time, dynamic, and technology-assisted intervention [40] can enhance patient engagement and capacity for self-management while reducing accessibility barriers faced by many vulnerable populations.

## Conclusions

This study demonstrated the effectiveness of a multidimensional digitised lifestyle intervention for the improvement of quality of life among women previously treated for blood, breast, and gynaecological cancers. The intervention employed multiple strategies for tailoring the intervention for individuals, integrating feedback and support mechanisms, and adopting theory-based behaviour change models. The complex and synergistic effects of many modifiable health behaviours emphasise the need for bundled health behaviour interventions to optimise women’s health and wellbeing after completion of active cancer treatment.

## Supplementary Information

**Additional file 1: Table S1.** Missing data and the ability to estimate primary endpoints. **Table S2.** Sample characteristics by baseline data completion^a^. **Table S3.** Between-group differences in SF-36 domain and total scores using intent-to-treat analysis. **Table S4.** Within-group changes in SF-36 domain and composite summary scores over time using intent-to-treat analysis^a^. **Table S5.** Comparisons of model fit for SF-36 data.

## Data Availability

The datasets generated and/or analysed during the current study are not publicly available but are available from the corresponding author on reasonable request.

## Abbreviations

WWACP: Women’s Wellness After Cancer Program
HRQoL: Health-related quality of life
RCT: Randomized controlled trial
IARC: International Agency for Research on Cancer
SF-36: Short Form Health Survey
FACT-G: Functional Assessment of Cancer Therapy-General
PF: Physical functioning
RP: Role limitations due to physical health
BP: Bodily pain
GH: General health
VT: Vitality
SF: Social functioning
RE: Role limitations due to emotional health
MH: Mental health
MCS: Mental Component Summary
PCS: Physical Health Component
SPSS: Statistical Package for the Social Sciences
PP: Per-protocol
ITT: Intent-to-treat
LTFU: Lost to follow-up
LMM: Linear mixed-effects models
AR: Autoregressive
AIC: Akaike Information Criterion
LR: Likelihood ratio
NHMRC: National Health and Medical Research Council

## References

[1] Sung H, Ferlay J, Siegel RL, Laversanne M, Soerjomataram I, Jemal A, Bray F (2021). Global Cancer Statistics 2020: GLOBOCAN estimates of incidence and mortality worldwide for 36 Cancers in 185 countries. CA Cancer J Clin.

[2] Ferlay J, Laversanne M, Ervik M, Lam F, Colombet M, Mery L, et al. Global Cancer Observatory: cancer tomorrow [https://gco.iarc.fr/tomorrow] Lyon: International Agency for Research on Cancer. 2020.

[3] Australian Institute of Health and Welfare (AIHW) (2019). Cancer in Australia 2019. Cancer series no 119.

[4] Thomaier L, Jewett P, Brown K, Gotlieb R, Teoh D, Blaes AH, Argenta P, Vogel RI (2020). The associations between physical activity, neuropathy symptoms and health-related quality of life among gynecologic cancer survivors. Gynecol Oncol.

[5] Wirtz P, Baumann FT (2018). Physical activity, exercise and breast Cancer - what is the evidence for rehabilitation, aftercare, and survival a review. Breast Care.

[6] Pennington KP, McTiernan A (2018). The role of physical activity in breast and gynecologic cancer survivorship. Gynecol Oncol.

[7] de Rooij BH, Thomas TH, Post KE, Flanagan J, Ezendam NP, Peppercorn J, Dizon DS (2018). Survivorship care planning in gynecologic oncology—perspectives from patients, caregivers, and health care providers. J Cancer Surviv.

[8] DeGuzman PB, Vogel DL, Horton B (2022). Examination of a distress screening intervention for rural cancer survivors reveals low uptake of psychosocial referrals. J Cancer Surviv..

[9] Nurnazahiah A, Shahril MR, Nor Syamimi Z, Ahmad A, Sulaiman S, Lua PL (2020). Relationship of objectively measured physical activity and sedentary behaviour with health-related quality of life among breast cancer survivors. Health Qual Life Outcomes.

[10] Chung SY, Oh J, Chang JS, Shin J, Kim KH, Chun K-H, Keum KC, Suh C-O, Kang S-M, Kim YB (2021). Risk of cardiac disease in patients with breast cancer: impact of patient-specific factors and individual heart dose from three-dimensional radiation therapy planning. Int J Radiat Oncol Biol Phys.

[11] Gallegos-Kearin V, Mix J, Knowlton S, Schneider JC, Zafonte R, Goldstein R (2016). Outcome trends of adult cancer patients receiving inpatient rehabilitation: a 10-year review of the uniform data system for medical rehabilitation. Arch Phys Med Rehabil.

[12] Smith SR, Zheng JY, Silver J, Haig AJ, Cheville A (2020). Cancer rehabilitation as an essential component of quality care and survivorship from an international perspective. Disabil Rehabil.

[13] Koll TT, Semin JN, Grieb BM, Dale W (2017). Motivating older adults with Cancer to keep moving: the implications of lifestyle interventions on physical activity. Curr Oncol Rep.

[14] Sallis JF, Floyd MF, Rodríguez DA, Saelens BE (2012). Role of built environments in physical activity, obesity, and cardiovascular disease. Circulation.

[15] WHO (2003). Adherence to long-term therapies. Evidence for action.

[16] Breast Cancer Network Australia. The financial impact of breast cancer. BCNA. n.d.

[17] Clinical Oncology Society of Australia Model of Survivorship Care Working Group. Model of survivorship care: critical components of cancer survivorship care in Australia Position Statement. In Clinical Oncology Society of Australia. 2016.

[18] Beesley VL, Alemayehu C, Webb PM (2018). A systematic literature review of the prevalence of and risk factors for supportive care needs among women with gynaecological cancer and their caregivers. Support Care Cancer.

[19] Australian Institute of Health and Welfare (AIHW) (2020). Rural and Remote Health.

[20] Anderson D, Seib C, Tjondronegoro D, Turner J, Monterosso L, McGuire A, Porter-Steele J, Song W, Yates P, King N (2017). The Women’s wellness after cancer program: a multisite, single-blinded, randomised controlled trial protocol. BMC Cancer.

[21] McLeod AI (1985). Remark AS R58: a remark on algorithm AS 183. An efficient and portable pseudo-random number generator. J J R Stat Soc Ser C Appl Stat.

[22] Wichmann B, Hill D (1982). Algorithm AS 183: an efficient and portable pseudo-random number generator. J R Stat Soc Ser C Appl Stat.

[23] Bandura A (2005). The primacy of self-regulation in health promotion. Appl Psychol.

[24] Cella DF, Tulsky DS, Gray G, Sarafian B, Linn E, Bonomi A, Silberman M, Yellen SB, Winicour P, Brannon J (1993). The functional assessment of Cancer therapy scale: development and validation of the general measure. J Clin Oncol.

[25] Ware J, Sherbourne C (1992). The MOS 36-Item Short-Form Health Survey (SF-36®): I. conceptual framework and item selection. Med Care.

[26] Chopra I, Kamal KM (2012). A systematic review of quality of life instruments in long-term breast cancer survivors. Health Qual Life Outcomes.

[27] Luckett T, King MT, Butow PN, Oguchi M, Rankin N, Price MA, Hackl NA, Heading G (2011). Choosing between the EORTC QLQ-C30 and FACT-G for measuring health-related quality of life in cancer clinical research: issues, evidence and recommendations. Ann Oncol.

[28] McHorney CA, Ware JE, Raczek AE (1993). The MOS 36-Item Short-Form Health Survey (SF-36): II. Psychometric and clinical tests of validity in measuring physical and mental health constructs. Med Care.

[29] Gupta SK (2011). Intention-to-treat concept: A review. Perspect Clin Res.

[30] Cohen J (1988). Statistical power analysis for the behavioral sciences.

[31] Gelman A (2006). Multilevel (hierarchical) modeling: what it can and cannot do. Technometrics.

[32] Bell K (2012). Remaking the self: trauma, teachable moments, and the biopolitics of cancer survivorship. Cult Med Psychiatry.

[33] Bluethmann SM, Vernon SW, Gabriel KP, Murphy CC, Bartholomew LK (2015). Taking the next step: a systematic review and meta-analysis of physical activity and behavior change interventions in recent post-treatment breast cancer survivors. Breast Cancer Res Treat.

[34] Stull VB, Snyder DC, Demark-Wahnefried W (2007). Lifestyle interventions in cancer survivors: designing programs that meet the needs of this vulnerable and growing population. J Nutr.

[35] Ghavami H, Akyolcu N (2017). The impact of lifestyle interventions in breast Cancer women after completion of primary therapy: a randomized study. J Breast Health.

[36] Anderson DJ, Seib C, McCarthy AL, Yates P, Porter-Steele J, McGuire A, Young L (2015). Facilitating lifestyle changes to manage menopausal symptoms in women with breast cancer: a randomized controlled pilot trial of The Pink Women’s Wellness Program. Menopause.

[37] Arun B, Austin T, Babiera GV, Basen-Engquist K, Carmack CL, Chaoul A, Cohen L, Connelly L, Haddad R, Harrison C (2016). A comprehensive lifestyle randomized clinical trial: design and initial patient experience. Integr Cancer Ther.

[38] Rosenman R, Tennekoon V, Hill LG (2011). Measuring bias in self-reported data. Int J Behav Healthc Res.

[39] Alfano CM, Ganz PA, Rowland JH, Hahn EE (2012). Cancer survivorship and cancer rehabilitation: revitalizing the link. J Clin Oncol.

[40] Penedo FJ, Oswald LB, Kronenfeld JP, Garcia SF, Cella D, Yanez B (2020). The increasing value of eHealth in the delivery of patient-centred cancer care. Lancet Oncol.

