# Supplementary material for: Improving health-related quality of life in women with breast, blood, and gynaecological Cancer with an eHealth-enabled 12-week lifestyle intervention: the women’s wellness after Cancer program randomised controlled trial
Source: BMC Cancer. 2022 Jul 8;22:747. doi: 10.1186/s12885-022-09797-6 (PMC9264489; doi:10.1186/s12885-022-09797-6)
Supplement: Supplementary file 1 — Additional file 1: Table S1. Missing data and the ability to estimate primary endpoints. Table S2. Sample characteristics by baseline data completiona. Table S3. Between-group differences in SF-36 domain and total scores using intent-to-treat analysis. Table S4. Within-group changes in SF-36 domain and composite summary scores over time using intent-to-treat analysisa. Table S5. Comparisons of model fit for SF-36 data. [file 12885_2022_9797_MOESM1_ESM.docx]

**Improving Health-related Quality of Life in Women with Breast, Blood, and Gynaecological** **Cancer with an eHealth-enabled 12-week Lifestyle Intervention: The Women’s Wellness after Cancer Program Randomised Controlled Trial**

Charrlotte Seib^1^, Debra Anderson^2^, Amanda McGuire^1^, Janine Porter-Steele^3, 4^, Nicole McDonald^5^, Sarah Balaam^4^, Diksha Sapkota^1^, Alexandra L. McCarthy^4,2,6^

^1^ Menzies Health Institute Queensland and School of Nursing and Midwifery, Griffith University, Queensland, Australia

^2^ Faculty of Health, University of Technology Sydney, New South Wales, Australia

^3^ Choices Cancer Support Program, Wesley Hospital, Brisbane, Queensland, Australia

^4^ School of Nursing, Midwifery and Social Work, The University of Queensland, and Mater Research Institute, Queensland, Australia

^5^ Menzies Health Institute Queensland, Griffith University, Queensland, Australia

^6^ School of Nursing, University of Auckland, New Zealand

Corresponding author: Debra Anderson, Faculty of Health, University of Technology Sydney, PO Box 123, Broadway, Sydney NSW, Australia; e-mail: [debra.anderson@uts.edu.au](mailto:debra.anderson@uts.edu.au%20)

**Table S1** Missing data and the ability to estimate primary endpoints

| Instruments | No. missing domain/summary scores ^a^ | | |
| --- | --- | --- | --- |
|  | *t_0_* | *t_1_* | *t_2_* |
| SF-36 domain sub-scales |  |  |  |
| Physical Functioning (PF) | 2 | 105 | 108 |
| Bodily Pain (BP) | 2 | 105 | 108 |
| General Health (GH) | 2 | 105 | 108 |
| Vitality (VT) | 2 | 106 | 108 |
| Social Functioning (SF) | 2 | 105 | 108 |
| Mental Health (MH) | 3 | 106 | 108 |
| Role limitations due to physical health (RP) | 7 | 107 | 111 |
| Role limitations due to emotional health (RE) | 8 | 117 | 109 |
| SF-36 composite scores |  |  |  |
| Physical component summary (PCS) ^b^ | 4 | 106 | 108 |
| Mental component summary (MCS ^b^ | 5 | 106 | 108 |

^a^ Missing scores reflect both loss to follow-up (LTFU) and missing values

^b^ PCS and MCS scores are calculated in participants who have available domain scores for at least seven of the eight sub-scales, although PCS cannot not be estimated without the PF scale and MCS cannot be estimated without the MH scale.

**Table S2** Sample characteristics by baseline data completion ^a^

|  | Complete baseline data | |
| --- | --- | --- |
|  | No (n = 68) | Yes (n = 283) |
|  | n (%) or M (SD) | n (%) or M (SD) |
| Socio-demographic characteristics |  |  |
| Mean Age (SD) | 53.0 (9.6) | 53.2 (8.6) |
| Marital status |  |  |
| Married/de facto relationship | 50 (74.6) | 216 (77.4) |
| Separated or divorced | 7 (10.4) | 32 (11.5) |
| Widowed | 3 (4.5) | 6 (2.2) |
| Single | 7 (10.4) | 25 (9.0) |
| Country of birth |  |  |
| Australia | 55 (83.3) | 187 (66.5)** |
| Elsewhere | 11 (16.7) | 94 (33.5) |
| Language other than English spoken at home |  |  |
| Yes | 12 (18.2) | 36 (10.4)* |
| No | 54 (81.8) | 309 (89.6) |
| Highest educational attainment |  |  |
| Year 10 or less (junior school) | 8 (12.1) | 23 (8.2) |
| Year 11 or 12 (senior school) | 9 (13.6) | 28 (10.0) |
| Technical certificate/diploma | 16 (24.2) | 63 (22.5) |
| University degree/postgraduate | 33 (50.0) | 166 (59.3) |
| Employment status |  |  |
| Employed (full-time) | 32 (50.0) | 122 (47.3) |
| Employed (part-time) | 22 (34.4) | 101 (39.1) |
| Retired | 7 (10.9) | 26 (10.1) |
| Other | 3 (4.7) | 9 (3.5) |
| Gross household income |  |  |
| Less than $20,000 AUD | 1 (1.5) | 5 (1.8)* |
| $20,000 - $80,000 AUD | 21 (31.8) | 79 (28.3) |
| Above $80,000 AUD | 36 (54.5) | 187 (67.0) |
| Don’t Know | 8 (12.1) | 8 (2.9) |
| Health-related quality of life |  |  |
| Mean PF (SD) | 45.9 (8.5) | 46.2 (9.2) |
| Mean RP (SD) | 43.0 (11.8) | 45.2 (12.1) |
| Mean BP (SD) | 47.6 (9.0) | 49.4 (9.1) |
| Mean GH (SD) | 47.5 (8.8) | 48.0 (9.3) |
| Mean VT (SD) | 46.4 (9.1) | 48.2 (9.9) |
| Mean SF (SD) | 45.1 (10.1) | 48.0 (8.5)* |
| Mean RE (SD) | 45.1 (12.1) | 47.1 (12.1) |
| Mean MH (SD) | 45.0 (9.3) | 46.4 (8.8) |
| Mean PCS (SD) | 46.4 (9.2) | 47.4 (9.2) |
| Mean MCS (SD) | 45.4 (10.8) | 47.4 (9.6) |

n, number of participants per group; M, mean value; SD, standard deviation of the mean value; AUD, Australian dollars; PF, Physical Functioning; RP, Role limitations due to physical health; BP, Bodily Pain; GH, General Health; VT, Vitality; SF, Social Functioning; RE, Role limitations due to emotional health; MH, Mental Health; PCS, Physical Component Summary; MCS, Mental Component Summary.

^a^ Overall n’s might differ because of missing data

* *p* <.05 ** *p* <.01

**Table S3** Between-group differences in SF-36 domain and total scores using intent-to-treat analysis

|  |  | Intervention group |  |  | Control group |  |
| --- | --- | --- | --- | --- | --- | --- |
|  | *t_0_* | *t_1_* | *t_2_* | *t_0_* | *t_1_* | *t_2_* |
| Variables | M(SD) | M(SD) | M(SD) | M(SD) | M(SD) | M(SD) |
| Physical Functioning (PF) | 47.0 (8.2) | 48.1 (8.7) | 48.8 (8.8) | 45.1 (9.0) | 46.6 (8.6) | 46.8 (9.1) |
| Role limitation/Physical health (RP) | 44.7 (11.7) | 47.9 (11.0) | 47.5 (11.1) | 42.3 (12.0) | 45.0 (12.1) | 44.5 (12.4) |
| Bodily Pain (BP) | 48.8 (9.1) | 50.1 (9.3)* | 49.7 (9.5) | 46.9 (8.9) | 47.1 (9.5)* | 47.3 (10.0) |
| General Health (GH) | 48.0 (8.3) | 48.0 (8.5) | 49.0 (9.1)* | 47.3 (9.5) | 46.5 (9.2) | 46.7 (10.0)* |
| Vitality (VT) | 47.0 (8.8) | 49.3 (8.8)* | 49.5 (9.3)* | 46.1 (9.8) | 47.0 (9.8)* | 47.1 (10.2)* |
| Social Functioning (SF) | 46.4 (9.2) | 48.5 (8.7) | 48.9 (9.0) | 44.7 (10.5) | 46.9 (10.3) | 47.3 (10.7) |
| Role limitations/emotional health (RE) | 45.8 (12.0) | 45.6 (11.9)** | 46.9 (11.5) | 45.6 (12.2) | 48.8 (10.7)** | 47.6 (11.9) |
| Mental Health (MH) | 45.3 (9.0) | 43.5 (8.9) | 48.9 (9.2)* | 45.2 (9.4) | 42.2 (9.5) | 47.0 (10.5)* |
| Physical Component Summary (PCS) | 47.8 (8.7) | 50.3 (9.2)* | 49.1 (9.4) | 45.4 (9.6) | 46.9 (9.4)* | 46.1 (10.0) |
| Mental Component Summary (MCS) | 45.7 (10.2) | 45.2 (10.0) | 48.4 (10.1) | 45.9 (10.8) | 45.9 (10.1) | 47.8 (11.3) |

*t_0_*_,_ baseline; *t_1_*_,_ 12-weeks (post-intervention); *t_2_*_,_ 24-weeks (follow-up); M, mean value; SD, standard deviation of the mean value; SF-36, short form 36; Intent-to-treat analysis using last number carried forward imputation method.

Between-group differences at *t_1_* and *t_2_* used one-way ANCOVA adjusting for baseline scores * *p* <.05 ** *p* <.01

**Table S4** Within-group changes in SF-36 domain and composite summary scores over time using intent-to-treat analysis ^a^

|  | n | M (SD) | | | Cohen’s d | |
| --- | --- | --- | --- | --- | --- | --- |
|  | *p_1_/p_2_* | *t_0_* | *t_1_* | *t_2_* | *d_1_* | *d_2_* |
| **Intervention group** |  |  |  |  |  |  |
| Physical functioning (PF) | 174/175 | 47.0 (8.2) | 48.1 (8.7)* | 48.9 (8.8)* | -0.17 | -0.18 |
| Role limitation/Physical health (RP) | 170/174 | 44.7 (11.6) | 48.0 (11.0)** | 47.5 (11.0) | -0.36 | 0.06 |
| Bodily pain (BP) | 174/175 | 48.9 (9.0) | 50.2 (9.2)* | 49.7 (9.3) | -0.18 | 0.08 |
| General health (GH) | 174/175 | 48.0 (8.3) | 48.1 (8.5) | 49.0 (9.0)* | -0.01 | -0.16 |
| Vitality (VT) | 174/175 | 47.2 (8.8) | 49.4 (9.0)** | 49.5 (9.4) | -0.30 | -0.01 |
| Social Functioning (SF) | 174/175 | 46.5 (9.2) | 48.6 (8.7)** | 49.0 (8.9) | -0.32 | -0.06 |
| Role limitations/emotional health (RE) | 172/174 | 45.5 (12.1) | 45.6 (11.9) | 47.0 (11.4) | -0.01 | -0.13 |
| Mental health (MH) | 173/175 | 45.1 (9.1) | 43.3 (9.3)** | 48.7 (9.3)** | 0.27 | -0.82 |
| Physical component summary (PCS) | 172/174 | 47.9 (8.7) | 50.4 (9.2)** | 49.2 (9.3)** | -0.39 | 0.22 |
| Mental component summary (MCS) | 171/174 | 45.5 (10.3) | 44.9 (10.3) | 48.2 (10.2)** | 0.07 | -0.47 |
| **Control group** |  |  |  |  |  |  |
| SF-36 |  |  |  |  |  |  |
| Physical functioning (PF) | 175/176 | 45.0 (8.9) | 46.6 (8.5)** | 46.8 (9.1) | -0.26 | -0.06 |
| Role limitation/Physical health (RP) | 174/176 | 42.1 (12.0) | 45.1 (12.0)** | 44.7 (12.4) | -0.28 | 0.04 |
| Bodily pain (BP) | 175/176 | 47.0 (8.9) | 47.3 (9.4) | 47.4 (9.9) | -0.04 | -0.01 |
| General health (GH) | 175/176 | 47.3 (9.5) | 46.5 (9.2) | 46.7 (10.0) | 0.12 | -0.04 |
| Vitality (VT) | 175/176 | 46.3 (9.8) | 47.1 (9.8) | 47.3 (10.1) | -0.13 | -0.02 |
| Social Functioning (SF) | 175/176 | 44.9 (10.4) | 47.0 (10.2)** | 47.4 (10.6) | -0.28 | -0.05 |
| Role limitations/emotional health (RE) | 171/176 | 45.5 (12.2) | 48.8 (10.6)** | 47.5 (11.9) | -0.34 | 0.14 |
| Mental health (MH) | 175/176 | 45.4 (9.4) | 42.4 (9.5)** | 47.1 (10.5)** | 0.47 | -0.62 |
| Physical component summary (PCS) | 175/176 | 45.3 (9.5) | 46.9 (9.3)* | 46.3 (10.0) | -0.24 | 0.11 |
| Mental component summary (MCS) | 175/176 | 46.1 (10.8) | 46.1 (10.1) | 47.8 (11.2)** | -0.01 | -0.21 |

p_1_, Pair 1 (t_0_ vs. t_1_); p_2_, Pair 2 (t_1_ vs. t_2_); M, mean value; SD, standard deviation of the mean value; d_1_, effect size for pair 1; d_2_, effect size for pair 2; SF-36, Short Form 36.

^a^ Split sample paired sample t–tests * *p* <.05 ** *p* <.01

**Table S5** Comparisons of model fit for SF-36 data

|  | Model 1 | | Model 2 | | Model comparisons ^a^ | |
| --- | --- | --- | --- | --- | --- | --- |
|  | LR *χ2* (16) | AIC | LR *χ2* (18) | AIC | LR *χ2* (2) | *p* |
| Physical Functioning (PF) | -2636.79 | 5305.58 | -2499.38 | 5034.76 | 274.82 | <.001 |
| Role limitation/Physical health (RP) | -2841.71 | 5715.42 | -2773.07 | 5582.14 | 137.28 | <.001 |
| Bodily Pain (BP) | -2731.08 | 5494.15 | -2614.02 | 5264.05 | 234.11 | <.001 |
| General Health (GH) | -2692.29 | 5416.57 | -2528.66 | 5093.31 | 327.26 | <.001 |
| Vitality (VT) | -2723.79 | 5479.58 | -2600.69 | 5237.39 | 246.19 | <.001 |
| Social Functioning (SF) | -2747.68 | 5527.37 | -2640.24 | 5316.48 | 214.89 | <.001 |
| Role limitations/emotional health (RE) | -2854.07 | 5740.13 | -2800.52 | 5637.04 | 107.09 | <.001 |
| Mental Health (MH) | -2716.74 | 5465.47 | -2576.42 | 5188.84 | 280.64 | <.001 |
| Physical component summary (PCS) | -2717.61 | 5467.21 | -2569.84 | 5175.69 | 295.53 | <.001 |
| Mental component summary (MCS) | -2801.47 | 5634.94 | -2685.13 | 5406.27 | 232.67 | <.001 |

Model 1 (Random intercept with interaction model); Model 2 (Random intercept/slope with interaction model); AIC, Akaike information criterion; LR χ2, Likelihood-ratio chi-squared test.

^a^ The LR test statistic compared the relative fit of nested models (i.e., LR = 2(ll(Model 2)-ll(Model 1))) where the LR test is statistically significant, the less restrictive model is significantly better than the more restrictive model.
